# Synthesis of Zinc Oxide Doped Magnesium Hydrate and Its Effect on the Flame Retardant and Mechanical Properties of Polypropylene

**DOI:** 10.3390/polym15214248

**Published:** 2023-10-28

**Authors:** Xue Li, Hongbo Zhang, Xiaoyuan Liu, Zhihui Lv, Yankui Jin, Donghai Zhu, Li Dang

**Affiliations:** 1 School of Chemical Engineering, Qinghai University, Xining 810016, China; m18798332923@163.com (X.L.); 18845581363@163.com (H.Z.); yuanyuan189112@163.com (X.L.); qhulzh@126.com (Z.L.); j13897682057@outlook.com (Y.J.); 2 State Key Laboratory of Plateau Ecology and Agriculture, Qinghai University, Xining 810016, China; zhudonghai-2001@163.com

**Keywords:** zinc oxide, magnesium hydrate, polypropylene, flame retardant, mechanical properties

## Abstract

In this work, an effective flame retardant consisting of nanoscale zinc oxide doped on the surface of hexagonal lamellar magnesium hydrate (ZO@MH) has been successfully synthesized via a hydrothermal process. Approximately 3-methacryloxypropyltrimethoxysilane (KH-570) is chosen as a modifier of ZO@MH for the purpose of enhancing the interfacial interaction between ZO@MH and the polypropylene (PP) matrix and reducing the agglomeration of ZO@MH. Afterwards, ZO@MH and KH-570 modified ZO@MH (KZO@MH) filled PP (PP/ZO@MH and PP/KZO@MH) composites are respectively prepared via the melt blending method. The flame retardant and smoke suppression properties of PP/ZO@MH and PP/KZO@MH composites are estimated by a cone calorimetry test (CCT). The peak value of the heat release rate of the PP/40KZO@MH composite is 327.0 kW/m^2^, which is 6.1% and 31.2% lower than that of the PP/40ZO@MH and PP/40MH composites, respectively. The lowest peak values of CO and CO_2_ production, 0.008 and 0.62 g/s, also appeared in the PP/40KZO@MH composite, which are 11.1% and 10.1% lower than those of the PP/40ZO@MH composite. Analysis of char residues indicates that nanoscale ZO and modification of KH-570 improve the amount and quality of char residues, which should be the main reason for the good flame retardant and smoke suppression properties of KZO@MH. Impact strength and nominal strain at break results show that the PP matrix is toughened by ZO@MH rather than KZO@MH. Tensile properties and the quantitative interfacial interaction calculated by the Turcsányi equation both prove the reinforcement of KZO@MH on the PP matrix.

## 1. Introduction

As an environment-friendly, non-toxicity, and low-cost flame retardant, magnesium hydrate (MH) has been applied in many flammable polymer matrixes, such as polypropylene (PP) [1,2], polyvinyl chloride (PVC) [3,4], high density polyethylene (HDPE) [5,6], linear low-density polyethylene (LLDPE) [7,8], ethylene-vinyl acetate (EVA) [9,10], polyurethane (PU) [11,12], polystyrene (PS) [13,14], polyethylene terephthalate (PET) [15,16], silicone rubber (SR) [5,17], and so on. However, at the mercy of low flame retardant efficiency, almost all of the polymer/MH composites exhibit good flame-retardant properties with quite a high filler content of MH. As a result, the processability and mechanical performance of the composites are greatly sacrificed. In theory, MH acts as a flame retardant in three ways: (i) oxygen and flammable volatiles around combustion zones are diluted by plenty of vapor generated from the thermal decomposition of MH; (ii) a magnesium oxide-containing barrier layer on the surface of the polymer matrix is formed to hinder the transportation of heat and flammable volatiles; and (iii) the temperature of the combusted polymer matrix is lowered through the endothermic decomposition of MH. It is interesting that MH also plays a special role in some particular polymers. For example, the hydrogen chloride produced from the dehydrochlorination of PVC can be neutralized by MH or generated magnesium oxide, which is good for the smoke suppression of PVC composites.

Mixing with other kinds of flame retardants to obtain a synergistic effect is a useful method for reducing the mass loading of MH flame retardant. For example, carbon compounds like carbon microspheres [16], reduced graphene oxide [18], multiwalled carbon nanotubes [19], expandable graphite [12,20], carbon black [21], etc., phosphorous compounds including miciroencapsulated red phosphorous [22], tripheyl phosphate [23], DOPO [24], etc., boron compounds just like zinc borate [18], boron nitride [11], boron oxide and anhydrous borax [25], etc., minerals such as montmorillonoid [26], organically modified montmorillonite (OMMT) [27], sepiolite [28], etc. are all reported as synergists of MH. The flame retardant efficiency of MH is ingeniously improved via the introduction of different flame retardant mechanisms, such as free radical trapping and the catalytic charring effect. Adding aluminum hydroxide [29] or magnesium carbonate [30] with MH can also show a considerable synergistic flame retardant effect, which is ascribed to the different decomposition temperatures.

In addition to these, transition metal oxides, such as zinc oxide and molybdenum trioxide, have drawn more and more attention to the flame retardant and smoke suppression properties of polymer matrix. The flame retardancy and smoke suppression mechanisms of transition metal oxides are summarized as follows: (1) catalyzed and cross-linked char-forming by Lewis acid (such as molybdenum trioxide, ammonium octamolybdate, etc.); and (2) reduction and coupling char-forming by the low-valent compounds of polyvalent transition metal oxides (such as Cu_2_O, oxalates, and formates, etc.). It should be pointed out that the flame retardant effect of transition metal oxides is often poor when they are used alone [31,32,33]. The combination of MH and transition metal oxides should generate a synergetic effect in flame retardancy and smoke suppression since they have entirely different mechanisms. In fact, we have successfully synthesized a hierarchical magnesium hydrate doped with molybdenum trioxide nanoparticles (MO@MH) via a hydrothermal process, which performs excellently in flame retardancy and smoke suppression of flexible PVC (fPVC) [18]. However, the high price of molybdate or molybdenum-containing compounds greatly limits the large-scale application of MO@MH. Hence, for this time, zinc oxide, another kind of transition metal oxide, is chosen as a synergist of MH for flame retardant and smoke suppression of PP due to its low price and potential catalyzed and cross-linked char-forming effect.

In this study, nanoscale zinc oxide is successfully introduced on the surface of hexagonal lamellar MH to synthesize zinc oxide-doped MH (ZO@MH) via a hydrothermal route. The morphology, composition, and structure of ZO@MH are characterized by scanning electron microscopy (SEM), elemental mapping, and X-ray powder diffraction (XRD). Approximately 3-methacryloxypropyltrimethoxysilane (KH-570) modified ZO@MH (KZO@MH) is evaluated and characterized by water contact angle, SEM, elemental mapping, and X-ray photoelectron spectroscopy (XPS). Afterwards, MH, ZO@MH, or KZO@MH-filled PP (PP/ZO@MH and PP/KZO@MH) composites are respectively prepared via the melt blending method. The flame retardant and smoke suppression effects of ZO@MH and KZO@MH on PP are estimated by the cone calorimetry test (CCT). The char residues are analyzed by SEM and XPS for the sake of a possible condensed-phase flame retardant mechanism. At last, the mechanical properties of the composites are also tested via impact and tensile tests. The compatibility is observed by SEM, and the interfacial interaction is calculated by the linear fitting of the Turcsányi equation.

## 2. Experimental

### 2.1. Materials

Sodium hydroxide (AR) and magnesium chloride hexahydrate (AR) were purchased from Tianjin Kemiou Chemical Regent Co., Ltd. (Tianjin, China). Zinc chloride (AR) was purchased from Tianjin Kaitong Chemical Regent Co., Ltd. (Tianjin, China). Approximately 3-methacryloxypropyltrimethoxysilane (≥95%) was purchased from Dongguan Dinghai Plastic Chemical Co., Ltd. (Dongguan, China). Ethanol (AR) and acetic acid (AR) were purchased from Sinopharm Chemical Regent Co., Ltd. (Shanghai, China). Polypropylene (T30S, 0.90 cm^3^/g) was purchased from Maoming Petrochemical Co., Ltd. (Maoming, China). Antioxidant 1010 (95.0%) was purchased from Tokyo Chemical Industry (Tokyo, Japan). All other materials were commercially available and used as received, unless otherwise noted.

### 2.2. Preparation of ZO@MH

Firstly, a certain amount of magnesium chloride hexahydrate was dissolved in deionized water to prepare a 0.5 mol/L magnesium chloride aqueous solution. Then, a certain amount of sodium hydroxide was added to the solution (molar ratio of Mg^2+^:OH^−^ = 1:2) and stirred for 0.5 h. The obtained suspension was aged for 12 h for crystal growth of magnesium hydrate. After that, a certain volume (molar ratio of Zn:Mg = 1:5) of 0.1 mol/L zinc chloride aqueous solution was dropwise added to the magnesium hydrate slurry with mechanical stirring, and the mixed solution was transferred to an autoclave. After hydrothermal reaction for 20 h at 90 °C, a ZO@MH hybrid was synthesized.

### 2.3. Preparation of KH-570 Functionalized ZO@MH

A total of 5.00 g of ZO@MH was added into 100 mL of deionized water with magnetic stirring for 3 h in order to obtain a pre-dispersed slurry of ZO@MH. KH-570, ethanol, and deionized water were mixed in a volume ratio of 18:1:1 to accomplish the hydrolysis process [34,35]. The pH value was adjusted to 4~6 by acetic acid. After 3 h of hydrolysis, a certain amount of KH-570 solution (mass ratio of ZO@MH:KH-570 = 100:9) was added to the pre-dispersed slurry of ZO@MH, which had been heated to 50 °C. The modification reaction was continued under magnetic stirring at 30 rpm for 1 h. The product (KZO@MH) was cooled to room temperature, then filtered and washed with plenty of ethanol and deionized water successively to remove excessive KH-570. The sample was collected and dried at 60 °C for 12 h.

### 2.4. Preparation of PP Composites

All fillers were dried at 60 °C for more than 8 h to remove moisture, and then PP granules, MH, ZO@MH, KZO@MH, and antioxidant 1010 were mixed based on a certain percentage with an RM-200C torque rheometer (HAPRO, Harbin, China) at 190 °C with a rotor speed of 60 rpm for 15 min. The composition and nomenclature used for samples in this article are presented in Table 1. Film samples with different thicknesses were obtained by compression molding with an XH-406B press vulcanizer (Xihua, Dongguan, China) at 190 °C for 8 min without pressure and 7 min under a pressure of 15 MPa, respectively. Then, the film samples were cooled to room temperature at the same pressure for 5 min. All the samples were stored at room temperature (23 ± 2 °C).

### 2.5. Characterization

The morphologies of ZO@MH, KZO@MH, and char residues after CCT were examined using a Merlin Compact scanning electron microscope (Zeiss, Jena, Germany) equipped with an energy-dispersive X-ray spectrometer (EDS). All samples were adhered to a copper-conductive belt and sputter-coated with a conductive gold layer.

The X-ray powder diffraction (XRD) patterns were determined by an X’Pert X-ray spectrometer (Philips, Eindhoven, The Netherlands) using Cu Kα radiation with a tube voltage of 40 kV and a tube current of 35 mA.

Water contact angle was measured by an OCA50 contact angle goniometer (Dataphysics, Stuttgart, Germany) at room temperature.

X-ray photoelectron spectroscopy (XPS) was carried out using an ESCALAB Xi^+^ multifunction electron spectrometer (Thermo Scientific, Waltham, MA, USA) equipped with an Al Kα X-ray source. The XPS spectra were taken in the constant analyzer energy mode with a pass energy of 100 eV and an energy step size of 0.4 eV, and high-resolution XPS spectra of C1s and Si2p were also recorded with an energy step size of 0.125 eV.

Cone calorimetry tests (CCT) were performed with samples of 100 mm × 100 mm × 3.0 mm on a CCT cone calorimeter (Motis Fire Technology (China) Co. Ltd., Suzhou, China) on the basis of standard GB/T 16172-2007. All samples were wrapped in an aluminum foil layer and then horizontally irradiated at a heat flux of 50 kW/m^2^.

Tensile properties were measured at room temperature using a 104B-EX electronic universal testing machine (Shenzhen Wance Testing Machine Co., Ltd., Shenzhen, China) according to GB/T 1040.2-2006. Dumbbell-shaped specimens (1AB) with a length of 75 mm and a thickness of 2 mm were employed, and the test speed was 10 mm/min. The results of each measurement were the average value of six samples, at least.

Impact strength was also measured at room temperature with a 501J-4 instrumental plastic impact testing machine (Shenzhen Wance Testing Machine Co., Ltd., Shenzhen, China) according to GB/T 1043-2008. The size of the testing samples was 80 mm × 10 mm × 4.0 mm with a V-type groove in the middle part. The maximum energy of the pendulum hammer was 11 J. The results of each measurement were the average value of six samples, at least.

## 3. Results and Discussion

### 3.1. Characterization of ZO@MH

**Figure 1:**
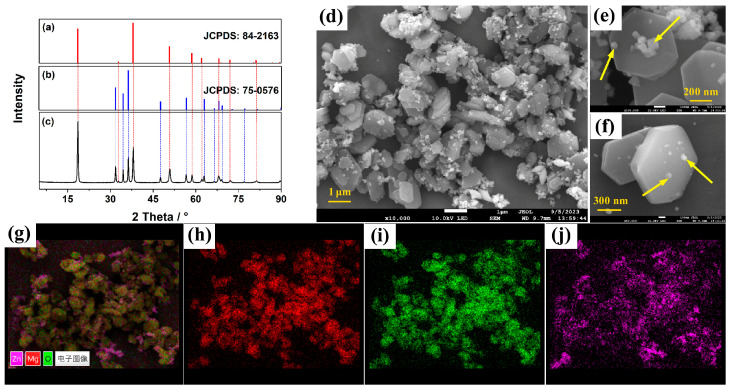
XRD patterns of MH (**a**), ZO (**b**), and ZO@MH (**c**); SEM images of ZO@MH (**d**–**f**); Element mappings (**g**) of ZO@MH: (**h**) for Mg, (**i**) for O, (**j**) for Zn.

The XRD patterns of ZO, MH, and ZO@MH are presented in Figure 1a–c. The uniform diffraction peaks at 2θ of 18.6°, 32.8°, 38.0°, 50.8°, 57.8°, 62.0°, 68.1°, 72.0°, and 80.3° are respectively assigned to (001), (100), (101), (102), (110), (111), (013), (021) and (022) crystal faces of MH (JCPDS: 84-2163) [36]. The peaks at 2θ of 31.8°, 34.5°, 36.3°, 47.7°, 56.7°, 63.0°, 66.5°, 68.1°, 69.3°, and 77.2° are respectively assigned to (100), (002), (101), (102), (110), (103), (200), (112), (201), and (202) crystal faces of ZO (JCPDS: 75-0576) [37]. As for ZO@MH, these diffraction peaks all appeared to have quite good fits. Hence, it is believed that the as-prepared sample contains MH and ZO. What more is, the high intensity of peaks and narrow peak widths both indicate the excellent crystallinity of ZO@MH. The detailed morphologies of ZO@MH are presented in Figure 1d–f. Obviously, ZO@MH is hierarchically constructed with hexagonal lamellar MH and nanoscale ZO particles on the surface. The length of the side and thickness of MH are about 1 μm and 100 nm, respectively. The size of ZO particles is about 50~100 nm, as pointed out by the yellow arrows in Figure 1e,f. In addition, the composition of ZO@MH is also explored using elemental mapping, as shown in Figure 1g–j. It is definitely true that ZO@MH is composed of Mg, Zn, and O elements (it also contains H elements, which cannot be detected by elemental mapping). The weight contents of Mg and Zn elements are 29.06 wt% and 14.68 wt%, respectively. Hence, the molar ratio of Mg:Zn is calculated as 5.3, which corresponds to the added ratio of Mg:Zn in the synthesis process.

### 3.2. Characterization of KZO@MH

**Figure 2:**
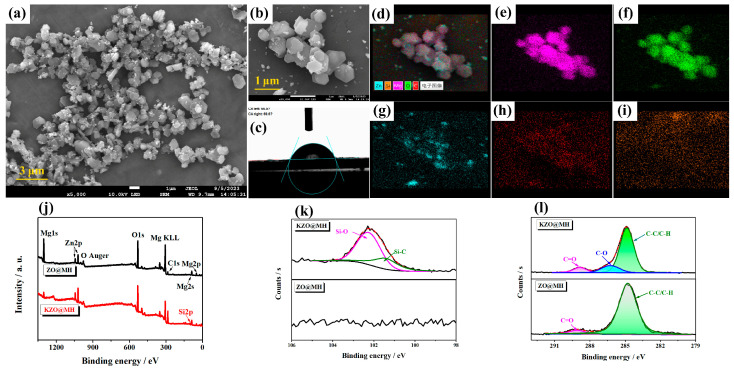
SEM (**a**,**b**) and water contact angle (**c**) images of KZO@MH; Element mappings (**d**) of ZO@MH: (**e**) for Mg, (**f**) for O, (**g**) for Zn, (**h**) for C, and (**i**) for Si; Full-scan XPS spectra (**j**), Si2p (**k**), and C1s (**l**) high-resolution spectra of ZO@MH and KZO@MH.

After modification with KH-570, KZO@MH maintains the same structure as ZO@MH, as shown in Figure 2a,b. The apparent hexagonal lamellar and nanoscale particles still exist, which not only indicates that KH-570 modification does not alter the morphologies of ZO@MH but also indicates that the degree of crystallinity of ZO@MH is not damaged during the modification process. However, the hydrophilicity surface of ZO@MH completely translates to a hydrophobicity surface of KZO@MH with a water contact angle of 69.9°, as shown in Figure 2c. The existence and distribution of KH-570 on the surface of KZO@MH are investigated by elemental mapping, as shown in Figure 2d–i. It can be inferred that KH-570 evenly coats the surface of ZO@MH because of the evenly distributed C element, as shown in Figure 2h. However, as shown in Figure 2i, the quite low content of Si (2.36 wt%) leads to an unconspicuous distribution compared with Figure 2d. The more detailed composition information, such as the varieties and states of different elements, is acquired by XPS. In Figure 2j, both ZO@MH and KZO@MH show several main character peaks located at 1304, 979, 533, 308, 285 Ev, 90 Ev, and 52 Ev, which were assigned to Mg1s, O Auger, O1s, Mg KLL, C1s, Mg2s, and Mg2p signals, respectively. The Si2p signal at 102 eV only appeared in the KZO@MH spectrum. The high-resolution spectrum of the Si2p signal of KZO@MH presented in Figure 2k can be split into two peaks, indicating two kinds of silicon-containing bonds in KZO@MH. The peak at 102.3 eV is assigned to the Si-O bond, which can be traced to the reaction between the OH- of ZO@MH and the Si-OH of KH-570. The peak at 101.5 eV is assigned to the Si-C bond, which comes from KH-570′s main chain [38]. In addition to these, the high-resolution spectra of C1s are markedly different between ZO@MH and KZO@MH. The appearance of the C-O signal at 286.2 eV and the decreasing percentage of the C-C/C-H signal at 284.8 eV both demonstrate the existence of KH-570 [39]. All these variations indicated that KH-570 is well bound to the surface of ZO@MH after surface modification.

### 3.3. Fire Hazards of PP Composites

#### 3.3.1. Fire Hazards Assessed by CCT

**Figure 3:**
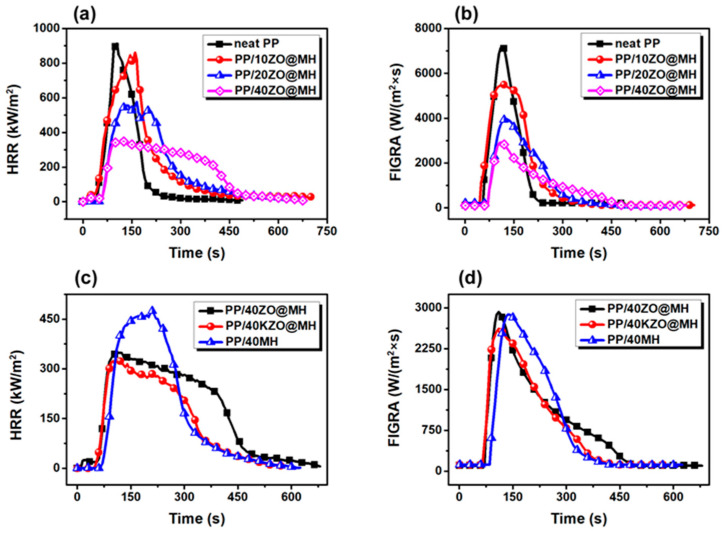
HRR (**a**,**c**) and FIGRA (**b**,**d**) curves of PP composites.

As is well known, CCT is probably the most important method for rapidly evaluating the flame retardant and smoke suppression properties of various materials. Figure 3 shows the heat release rate (HRR) and fire growth rate index (FIGRA) of neat PP and its composites at a heat flux of 50 kW/m^2^, and the corresponding data are listed in Table 2. A high and narrow HRR curve of neat PP is observed in Figure 3a, with the peak and average values of HRR (pHRR and avHRR) reaching 932.7 and 174.5 kW/m^2^, respectively, as shown in Table 2. The immediate burning of neat PP is effectively suppressed with the addition of ZO@MH. When 40 phr (parts per hundred) of ZO@MH is added, the pHRR and avHRR values are decreased to 348.4 and 163.9 kW/m^2^, respectively, which are 62.6% and 6.1% lower than those of neat PP. For comparison, 40 phr of KZO@MH or MH are respectively added to the PP matrix, and the HRR curves are presented in Figure 3c. Obviously, PP/40ZO@MH and PP/40KZO@MH composites show lower pHRR and avHRR values than those of PP/40MH composites, indicating the better flame retardancy of ZO@MH and KZO@MH than MH. What is more notable is that the KH-570 modification further improves the flame retardancy of ZO@MH. The lowest pHRR and avHRR of PP/40KZO@MH are 327.0 and 137.2 kW/m^2^, respectively, which are 6.1% and 16.3% lower than those of the PP/40ZO@MH composite. Considering the small amount of KH-570 in the modification process, the result is satisfactory. In our opinion, the KH-570 modification mainly ameliorates the agglomeration and interfacial compatibility issues of ZO@MH in the PP matrix. A well-distributed polymer/filler composite can perform the functions of fillers better than a PP/ZO@MH composite. In addition, the possible flame retardant synergism effect between ZO@MH and KH-570 is another non-negligible reason [17].

The tendency of FIGRA variations of neat PP and its composites is presented in Figure 3b,d. Broadly speaking, it is very similar to the tendency of HRR variations in Figure 3a,c. The peak values of FIGRA (pFIGRA) of PP composites decrease with the increasing ZO@MH amount. When 40 phr of ZO@MH is added, the pFIGRA value is decreased to 2928.7 W/(m^2^ × s), which is 59.4% lower than that of neat PP. In other words, fire growth is effectively inhibited by the addition of ZO@MH. In fact, the size and intensity of the flame for PP/ZO@MH composites are remarkably reduced, as observed during the test process. In addition, Figure 3d shows the superiority of KZO@MH in flame retardancy again. The lowest pFIGRA value of PP/40KZO@MH is 2621.2 W/(m^2^ × s), which is 63.6% and 10.5% lower than that of neat PP and PP/40ZO@MH composite, respectively.

**Figure 4:**
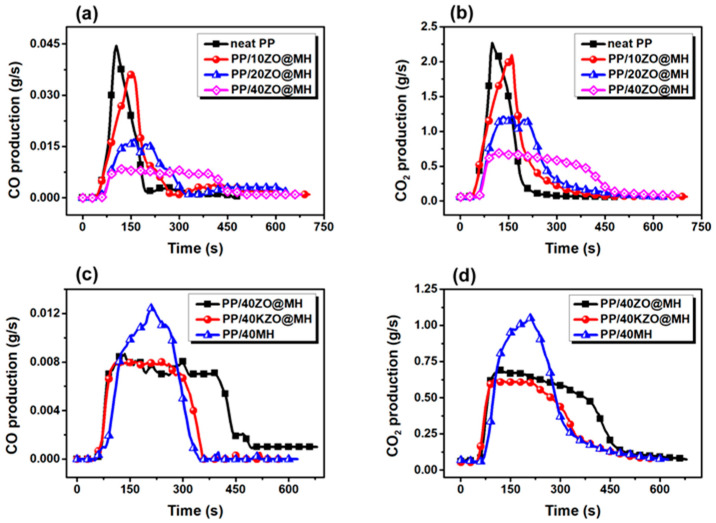
CO (**a**,**c**) and CO_2_ (**b**,**d**) production curves of PP composites.

Smoke, typified by CO and CO_2_, is another key issue during the burning process, since asphyxia is considered the main cause of fatalities in a real fire. As shown in Figure 4a,b, the peak values of CO and CO_2_ production (pCOP and pCO_2_P) of PP composites are both greatly lowered by the addition of ZO@MH, indicating the good smoke suppression effect of ZO@MH. As for the PP/40ZO@MH composite, the pCOP and pCO_2_P values are decreased to 0.009 and 0.69 g/s, respectively, which are 79.5% and 69.6% lower than those of neat PP. Hence, the smoke safety class of neat PP is significantly improved. It is worth mentioning that the pCOP and pCO_2_P values of the PP/40ZO@MH composite are also lower than those of the PP/40MH composite, as shown in Figure 4c,d and Table 2, indicating the importance of nanoscale ZO doping on the surface of MH. Last but not least, the lowest pCOP and pCO_2_P values, 0.008 and 0.62 g/s, also appeared in the PP/40KZO@MH composite, indicating the importance of KH-570 modification. The reduction of CO and CO_2_ production by ZO@MH can be attributed to the char-forming catalysis effect of nanoscale ZO [40]. However, the char residue of PP/40MH composite (34.93%) is even higher than that of PP/40ZO@MH composite (31.25%), as shown in Table 2, probably due to the restricted performance of unevenly distributed ZO@MH in the PP matrix.

#### 3.3.2. Analysis of Char Residue

**Figure 5:**
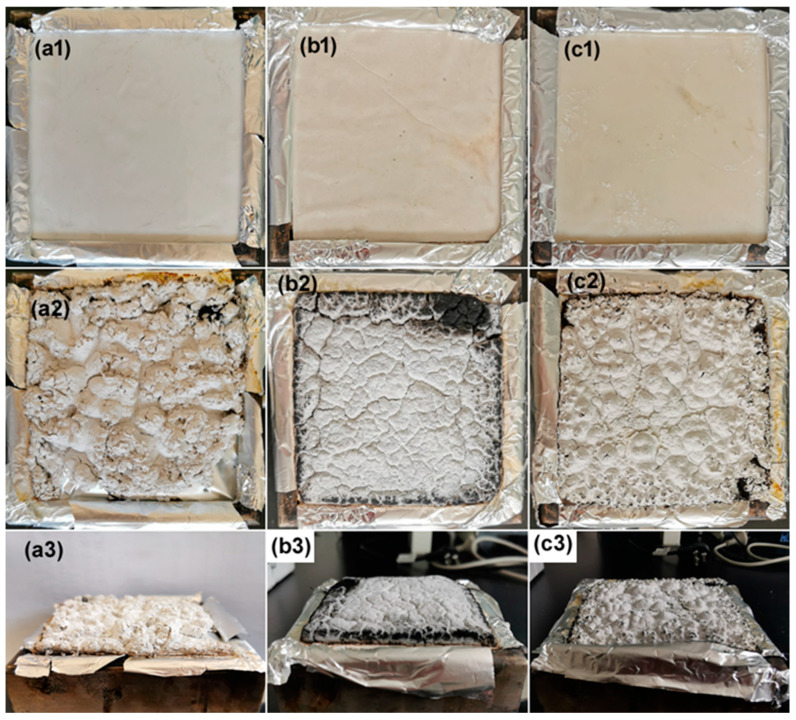
Photos of the char residues of PP/40MH (**a1**–**a3**), PP/40KZO@MH (**b1**–**b3**), and PP/40ZO@MH (**c1**–**c3**) composites before (serial number 1) and after (serial number 2 for top view and serial number 3 for side view) CCT test.

**Figure 6:**
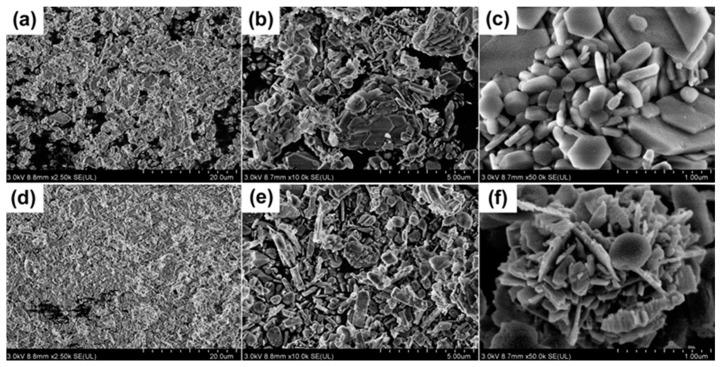
SEM images of char residues of PP/40KZO@MH (**a**–**c**) and PP/40ZO@MH (**d**–**f**) composites.

Not only the amount but also the quality of the char residue can greatly affect the flame retardant and smoke suppression effects of PP composites. As shown in Figure 5, PP/40MH, PP/40KZO@MH, and PP/40ZO@MH composites all turn into white char residues after burning. The strength of these char residues is quite low, so a finger touch caused the structure to crumble to dust, especially for the char residue of the PP/40MH composite. The top views show that PP/40KZO@MH char residue contains a relatively complete structure compared with the other two. Cracks and holes are more commonly seen in the char residues of PP/40MH and PP/40ZO@MH composites. The lateral views show that there is no expansion for the three kinds of char residues. However, bubble structures are observed on the surface of the char residues of PP/40MH and PP/40ZO@MH composites. It can be imagined that abundant vapor is generated in the thermal decomposition of MH in a very short time, and then a “boiling” phenomenon is formed, as for PP/40MH and PP/40ZO@MH composites. The flat structure of the char residue of the PP/40KZO@MH composite reveals the slower decomposition of KZO@MH than ZO@MH and MH. The more detailed information on the surface layer of char residues can be obtained from the SEM images in Figure 6. Both PP/40KZO@MH and PP/40ZO@MH composites show a loose-structured char residue, which dominates and contains decomposed MH and slightly carbon particles. The hexagonal lamellar MH (or MgO) is clearly seen in Figure 6c.

**Figure 7:**
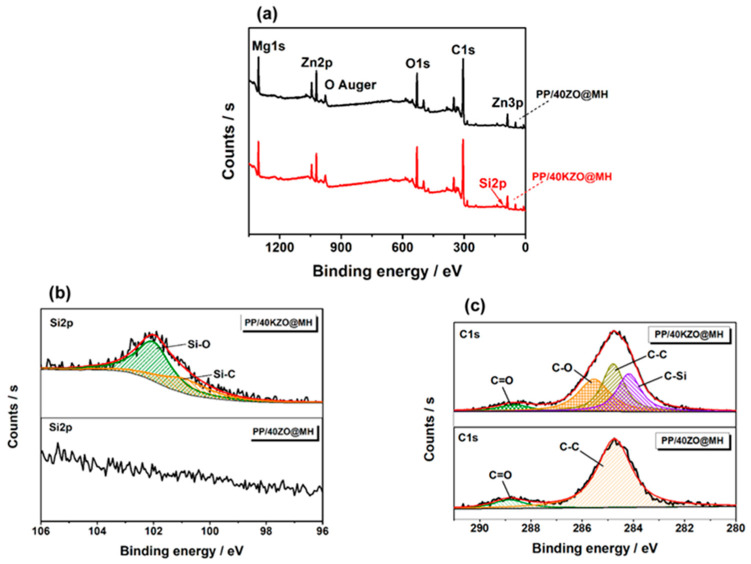
Full-scan XPS spectra (**a**), Si2p (**b**), and C1s (**c**) high-resolution spectra of char residues of PP/40ZO@MH and PP/40KZO@MH composites.

The inner layers of the char residues of PP/40KZO@MH and PP/40ZO@MH composites are stronger and harder than the outers, due to the higher content of carbons. In other words, ZO@MH and KZO@MH migrate to the surface of composites during the burning process and finally form a loose outer layer [41]. XPS is used to further analyze the varieties and states of different elements in the inner layers of the char residues of PP/40KZO@MH and PP/40ZO@MH composites. As shown in Figure 7a, the full-scan XPS spectra of char residues of PP/40KZO@MH and PP/40ZO@MH composites show several main character peaks located at 1304, 1021, 979, 533, 308, 285, 139, 88 eV, and 50 eV, which were assigned to Mg1s, Zn2p, O Auger, O1s, Mg KLL, C1s, Zn3s, Zn3p, and Mg2p signals, respectively. Si2p signal at 102 eV only appears in the char residue of the PP/40KZO@MH composite spectrum. The high-resolution spectrum presented in Figure 7b is split into two peaks, indicating two kinds of silicon-containing bonds in the char residue of the PP/40KZO@MH composite. The peak at 102.2 eV is also assigned to the Si-O bond, and the peak at 101.3 eV is also assigned to the Si-C bond. In addition to these, the high-resolution spectra of C1s are markedly different between the char residues of PP/40ZO@MH and PP/40KZO@MH composites. Except for the C=O signal at 288.8/288.7 eV and the C-C/C-H signal at 284.8 eV, the C-O signal at 285.6 eV and the C-Si signal at 284.2 eV only appeared in the high-resolution spectrum of C1s of the char residue of the PP/40KZO@MH composite. All these variations indicated that KH-570 deeply participates in the formation of the condensed phase and plays a vital role in the better flame retardant and smoke suppression effects.

### 3.4. Mechanical Properties of PP Composites

#### 3.4.1. Impact Properties

**Figure 8:**
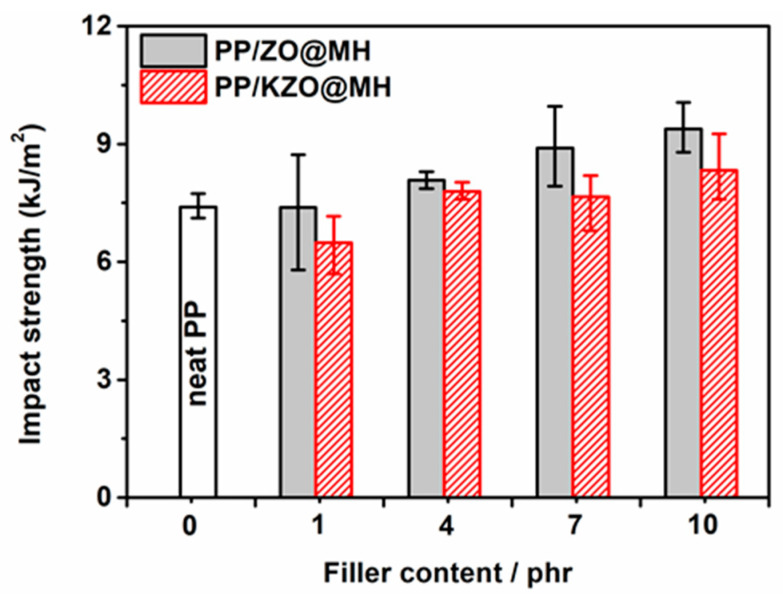
Impact strength of PP/ZO@MH and PP/KZO@MH composites.

**Figure 9:**
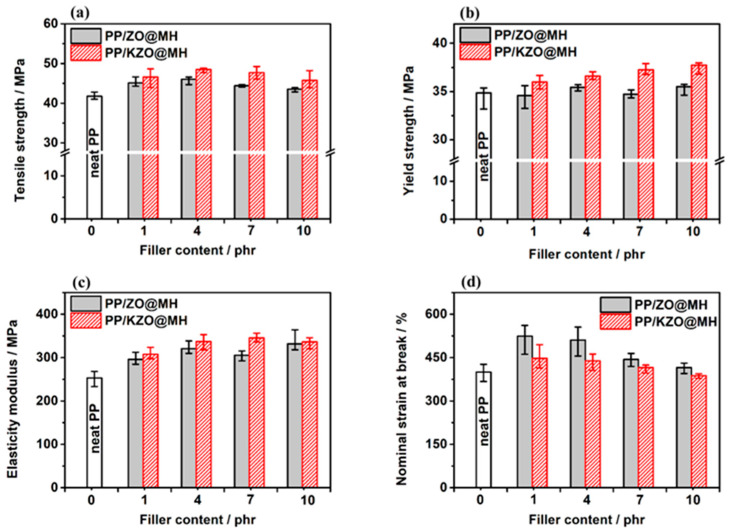
Tensile strength (**a**), yield strength (**b**), elasticity modulus (**c**), and nominal strains at break (**d**) of PP/ZO@MH and PP/KZO@MH composites.

Figure 8 shows the impact strength of PP/ZO@MH and PP/KZO@MH composites with different filler contents, and the corresponding data are summarized in Table 3. Firstly, an apparent increasing trend of impact strength is observed with the increasing ZO@MH. When 10 phr of ZO@MH is added, the impact strength of the PP/10ZO@MH composite reaches 9.38 kJ/m^2^, which is 26.9% higher than that of neat PP. Hence, the toughening effect of ZO@MH on the PP matrix is undeniable. Secondly, the impact strengths of PP/KZO@MH composites are always lower than those of PP/ZO@MH composites when filler content is the same. For example, the impact strength of PP/7KZO@MH composite is 7.67 kJ/m^2^, which is 13.8% lower than that of PP/7ZO@MH composite, as shown in Table 3. For the PP/1KZO@MH composite, the impact strength is only 6.49 kJ/m^2^, which is even 12.2% lower than that of neat PP. The introduction of KH-570 does not benefit the toughening effect of ZO@MH, probably due to the too strong interfacial interactions between the PP matrix and KZO@MH, which go against interfacial debonding and finally lead to brittle fracture of composites with an obvious decrease in impact strength. The possible conversion of different kinds of PP crystal (α, β, and γ) results from the introduction of ZO@MH or KZO@MH, which may be another factor in toughening [42]. In addition, the tensile toughness of PP/ZO@MH and PP/KZO@MH composites exhibits a similar trend, as shown in Figure 9d. When ZO@MH is replaced by the same amount of KZO@MH, the nominal strain at break of PP composites decreases by 14.6~6.7%, as shown in Table 3.

#### 3.4.2. Tensile Properties

**Figure 10:**
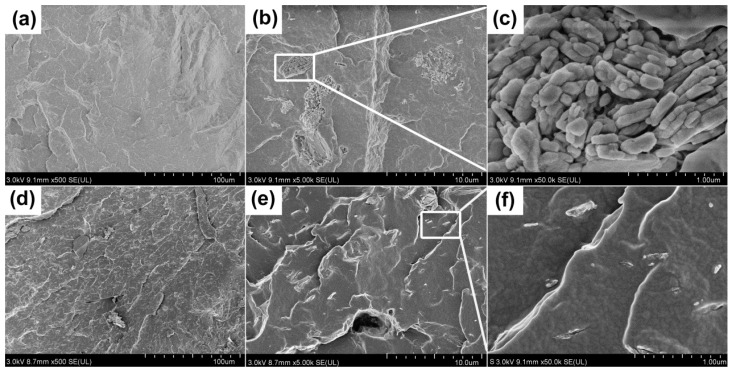
SEM images of PP/10ZO@MH (**a**–**c**) and PP/10KZO@MH (**d**–**f**) composites.

Figure 9 shows the tensile properties, such as tensile strength, yield strength, elasticity modulus, and nominal strain at break, of PP/ZO@MH and PP/KZO@MH composites with different filler content, and the corresponding data are also summarized in Table 3. From Figure 9a–c, the tensile strength, yield strength, and elasticity modulus of PP/ZO@MH composites are always improved with the substitution of ZO@MH by KZO@MH. For instance, the tensile strength, yield strength, and elasticity modulus of the PP/7KZO@MH composite are 47.71, 37.24, and 345.56 MPa, which are 7.38%, 7.28%, and 11.6% higher than those of the PP/7ZO@MH composite, as shown in Table 3. The remarkable reinforcement of KZO@MH on PP matrix can be attributed to two reasons: (i) better distribution and lesser agglomerations of KZO@MH, as proved by the SEM images of Figure 10, and (ii) stronger interfacial interactions between PP matrix and KZO@MH. On the one hand, KH-570 is well bound to the surface of ZO@MH after surface modification. On the other hand, the long chain structure, C=C, and C=O structures in KH-570 improve the interfacial compatibility and entanglement between the KZO@MH and PP matrix. As shown in Figure 10e,f, the interfaces between fillers (KZO@MH) and the PP matrix are blurry and unclear, which can effectively transfer the stress from the matrix to the fillers. The quantitative calculations of interfacial interaction are also executed by the famous Turcsányi equation [43,44].

(1) $$\sigma_{\mathrm{yc}}=\sigma_{\mathrm{yp}}\frac{1-\phi_{f}}{1+2.5\phi_{f}}\exp\left(B\phi_{f}\right)$$

where *σ*_yc_ and *σ*_yp_ are the yield strengths of composites and neat polymers, respectively. *ϕ*_f_ is the volume fraction of fillers in composites. *B* represents the strength of interfacial interactions. The bigger the value of *B*, the stronger the interfacial interactions between fillers and matrix. In general, Formula (1) can be converted into Formula (2) after the logarithm.

(2) $$\ln\left(\frac{\sigma_{\mathrm{yc}}}{\sigma_{\mathrm{yp}}}\right)+\ln\left(\frac{1+2.5\phi_{f}}{1-\phi_{f}}\right)=B\phi_{f}$$

**Figure 11:**
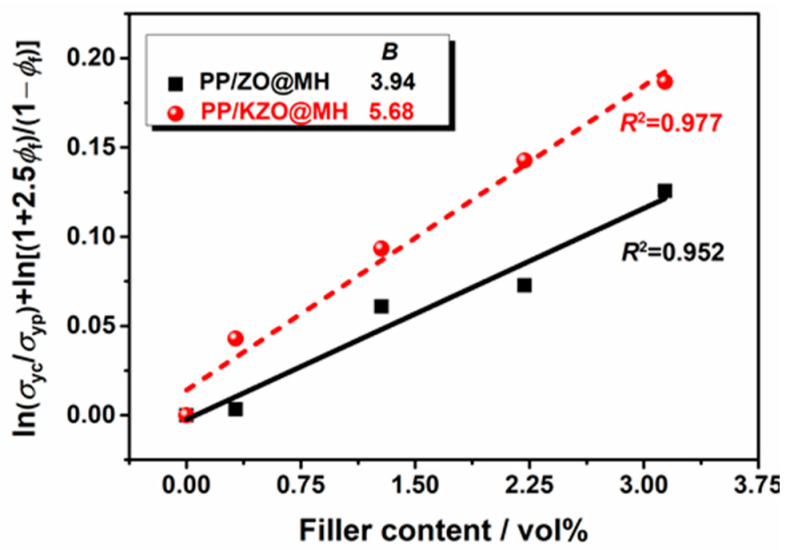
Linear fitting of the Turcsányi equation for PP composites filled with different amounts of ZO@MH and KZO@MH.

Figure 11 shows the linear fitting result of the Turcsányi equation for PP composites. It should be pointed out that the volume fractions of ZO@MH or KZO@MH are calculated by the apparent density of MH (2.36 g/cm^3^) and ZO (5.6 g/cm^3^) with the molar ratio of MH:ZO = 5.3:1. The mixing effect on volume and other capacity properties is not considered in this situation. As shown in Figure 11, the *B* values of PP/ZO@MH and PP/KZO@MH composites are calculated as 3.94 and 5.68, respectively, indicating stronger interfacial interactions between KZO@MH and the PP matrix. KH-570 acts as a bridge between inorganic ZO@MH and the PP matrix.

## 4. Conclusions

In this study, an effective flame retardant consisting of nanoscale zinc oxide doped on the surface of hexagonal lamellar magnesium hydrate (ZO@MH) has been successfully synthesized via a hydrothermal process. Approximately 3-methacryloxypropyltrimethoxysilane (KH-570) is chosen as a modifier of ZO@MH for the purpose of enhancing the interfacial interaction between ZO@MH and the polypropylene (PP) matrix and reducing the agglomeration of ZO@MH. Scanning electron microscopy (SEM) images, X-ray photoelectron spectroscopy (XPS), and water contact angle measurement results indicate that KH-570 modifications do not alter the morphologies of ZO@MH but covert the hydrophilicity surface of ZO@MH to a hydrophobicity surface of KZO@MH with a water contact angle of 69.9°. Afterwards, ZO@MH and KH-570 modified ZO@MH (KZO@MH) filled PP (PP/ZO@MH and PP/KZO@MH) composites are respectively prepared via the melt blending method. The peak value of heat release rate (pHRR) of PP/40KZO@MH is 327.0 kW/m^2^, which is 6.1% and 31.2% lower than that of PP/40ZO@MH and PP/40MH composites, respectively. The average value of heat release rate (avHRR) of the PP/40KZO@MH composite is 137.2 kW/m^2^, which is 16.3% and 10.7% lower than that of the PP/40ZO@MH and PP/40MH composites, respectively. The lowest peak values of CO and CO_2_ production (pCOP and pCO_2_P), 0.008 and 0.62 g/s, also appeared in the PP/40KZO@MH composite, which are 11.1% and 10.1% lower than those of the PP/40ZO@MH composite. In comparison with PP/40MH, the pCOP and pCO_2_P values of the PP/40KZO@MH composite are about 30~40% lowered, indicating an apparent advantage of KZO@MH on flame retardant and smoke suppressed PP matrix. Analysis of char residues indicates that nanoscale ZO and modification of KH-570 improve the amount and quality of char residues, which should be the main reason for the good flame retardant and smoke suppression properties of KZO@MH. When 10 phr of ZO@MH is added, the impact strength of the PP/10ZO@MH composite reaches 9.38 kJ/m^2^, which is 26.9% higher than that of neat PP. However, the impact strength of the PP/7KZO@MH composite is 7.67 kJ/m^2^, which is 13.8% lower than that of the PP/7ZO@MH composite. Tensile properties and the quantitative interfacial interaction calculated by the Turcsányi equation both prove the reinforcement of KZO@MH on the PP matrix. The remarkable reinforcement of KZO@MH on PP matrix can be attributed to two reasons: (i) better distribution and lesser agglomerations of KZO@MH, and (ii) stronger interfacial interactions between PP matrix and KZO@MH.

## Figures and Tables

**Table 1:** Composition and nomenclature used in this article.

Label	Filler Type	phr ^a^ of Filler
neat PP	/	*I* = 1, 4, 7, 10, 20, 40
PP/*i*MH	MH	
PP/*iZ*O@MH	ZO@MH	
PP/*i*K*Z*O@MH	KZO@MH	

^a^ parts per hundred.

**Table 2:** CCT data for neat PP and its composites.

Sample	pHRR (kW/m^2^)	avHRR (kW/m^2^)	pFIGRA (W/(m^2^ × s))	pCOP (g/s)	pCO_2_P (g/s)	Residue (wt%)
neat PP	932.7	174.5	7208.3	0.044	2.27	0.79
PP/10ZO@MH	862.3	183.8	5489.5	0.036	2.10	16.14
PP/20ZO@MH	577.5	182.3	3966.0	0.017	1.22	26.69
PP/40ZO@MH	348.4	163.9	2928.7	0.009	0.69	31.25
PP/40KZO@MH	327.0	137.2	2621.3	0.008	0.62	37.23
PP/40MH	475.1	153.7	2883.6	0.012	1.05	34.93

**Table 3:** Data on the tensile and impact properties of neat PP and its composites.

Sample	Impact Strength (kJ/m^2^)	Tensile Strength (MPa)	Yield Strength (MPa)	Elasticity Modulus (MPa)	Nominal Strains at Break (%)
neat PP	7.39	41.76	34.85	252.99	400.15
PP/1ZO@MH	7.39	45.13	34.57	295.86	524.20
PP/4ZO@MH	8.08	46.04	35.43	320.39	510.80
PP/7ZO@MH	8.90	44.43	34.72	305.62	443.74
PP/10ZO@MH	9.38	43.54	35.49	331.88	415.14
PP/1KZO@MH	6.49	46.58	35.97	307.50	447.44
PP/4KZO@MH	7.80	48.52	36.60	336.79	438.37
PP/7KZO@MH	7.67	47.71	37.24	345.56	415.56
PP/10KZO@MH	8.33	45.75	37.73	336.09	387.49

## Data Availability

The data presented in this study are available on request from the corresponding author.
